# Supplementary material for: The Systems Biology Research Tool: evolvable open-source software
Source: BMC Syst Biol. 2008 Jun 29;2:55. doi: 10.1186/1752-0509-2-55 (PMC2446383; doi:10.1186/1752-0509-2-55)
Supplement: Additional file 1 — SBRT Archive. An archive of the current version of the Systems Biology Research Tool. [file 1752-0509-2-55-S1.zip › sbrt-1.4.0/doc/developers_guide/api/sbrt/kernel/math/comb/proc/StrictSingleElementUnionProc.html]

StrictSingleElementUnionProc


|  |  |  |
| --- | --- | --- |
| **Overview** \| **Package** \| **Class** \| **Use** \| **Tree** \| **Deprecated** \| **Index** \| **Help** | |  |
| **PREV CLASS**   NEXT CLASS | **FRAMES**    **NO FRAMES**     **All Classes** |  |
| SUMMARY: NESTED \| FIELD \| CONSTR \| METHOD | DETAIL: FIELD \| CONSTR \| METHOD |  |


---


## sbrt.kernel.math.comb.proc Class StrictSingleElementUnionProc<E>

```
java.lang.Object
  sbrt.kernel.math.comb.proc.StrictSingleElementUnionProc<E>
```

**Type Parameters:**: `E` - the element type.

**All Implemented Interfaces:**: KernelProcess<java.util.Set<java.util.Set<E>>>

---

``` public class StrictSingleElementUnionProc<E> extends java.lang.Object implements KernelProcess<java.util.Set<java.util.Set<E>>> ```

This class is used to wrap the strict single-element union computer as a process.

**Author:**
:   This class was written and documented by Jeremiah Wright
    while in the Wagner lab.

---

**Constructor Summary**

`StrictSingleElementUnionProc()`
  
          Constructs a new strict single-element union process.


| **Method Summary** | |
| --- | --- |
| `void` | `run(ResultsManager<java.util.Set<java.util.Set<E>>> resultsManager)`             Computes the strict single-element unions of the provided collection of sets, and adds the results to the provided results manager. |
| `void` | `setSets(java.util.Collection<java.util.Set<E>> sets)`             Sets the collection of sets to that provided. |

**Methods inherited from class java.lang.Object**

`clone, equals, finalize, getClass, hashCode, notify, notifyAll, toString, wait, wait, wait`

**Constructor Detail**

### StrictSingleElementUnionProc

```
public StrictSingleElementUnionProc()
```

:   Constructs a new strict single-element union process.


**Method Detail**

### setSets

```
public void setSets(java.util.Collection<java.util.Set<E>> sets)
```

:   Sets the collection of sets to that provided.

    :   **Parameters:**: `sets` - a collection of sets of elements.

---


### run

```
public void run(ResultsManager<java.util.Set<java.util.Set<E>>> resultsManager)
```

:   Computes the strict single-element unions of the provided collection of sets,
    and adds the results to the provided results manager.

    :   **Specified by:**: `run` in interface `KernelProcess<java.util.Set<java.util.Set<E>>>`
    :   **Parameters:**: `resultsManager` - the results manager to which the computed results are to be added.


---


|  |  |  |
| --- | --- | --- |
| **Overview** \| **Package** \| **Class** \| **Use** \| **Tree** \| **Deprecated** \| **Index** \| **Help** | |  |
| **PREV CLASS**   NEXT CLASS | **FRAMES**    **NO FRAMES**     **All Classes** |  |
| SUMMARY: NESTED \| FIELD \| CONSTR \| METHOD | DETAIL: FIELD \| CONSTR \| METHOD |  |


---
